# Cycling in a crowd: Coordination of plant cell division, growth, and cell fate

**DOI:** 10.1093/plcell/koab222

**Published:** 2021-09-08

**Authors:** Robert Sablowski, Crisanto Gutierrez

**Affiliations:** Cell and Developmental Biology, John Innes Centre, Norwich, UK; Centro de Biología Molecular Severo Ochoa, CSIC-UAM, Nicolas Cabrera 1, Cantoblanco, 28049 Madrid, Spain

## Abstract

The reiterative organogenesis that drives plant growth relies on the constant production of new cells, which remain encased by interconnected cell walls. For these reasons, plant morphogenesis strictly depends on the rate and orientation of both cell division and cell growth. Important progress has been made in recent years in understanding how cell cycle progression and the orientation of cell divisions are coordinated with cell and organ growth and with the acquisition of specialized cell fates. We review basic concepts and players in plant cell cycle and division, and then focus on their links to growth-related cues, such as metabolic state, cell size, cell geometry, and cell mechanics, and on how cell cycle progression and cell division are linked to specific cell fates. The retinoblastoma pathway has emerged as a major player in the coordination of the cell cycle with both growth and cell identity, while microtubule dynamics are central in the coordination of oriented cell divisions. Future challenges include clarifying feedbacks between growth and cell cycle progression, revealing the molecular basis of cell division orientation in response to mechanical and chemical signals, and probing the links between cell fate changes and chromatin dynamics during the cell cycle.

## Teaser

Plant cell cycle and division are linked to specific cell fates and respond to growth-related cues, such as metabolic state, cell size, cell shape, and mechanical stress.

## Introduction

Cell cycle progression and cell division are core processes for life, directly connected with the replication of genetic material and its transmission through generations. Accordingly, many aspects of the cell cycle machinery are conserved and likely inherited from the common unicellular ancestors of all eukaryotes. Superimposed on these ancient mechanisms, multicellularity required the evolution of mechanisms that coordinate the growth and proliferation of cells across developing organs and with the acquisition of specialized cell fates.

Multicellularity evolved separately in plants, so the conserved core regulators of cell cycle progression are linked to morphogenesis and cell fate by plant-specific pathways. Furthermore, particular features of plant cells, such as the presence of interconnected cell walls, have important consequences for the mechanism of cell division and for the coordination of growth and division across tissues. Plant development is largely postembryonic, strictly depends on the continuous supply of new cells to form organ primordia and cannot rely on cell migration. For these reasons, plant morphogenesis depends chiefly on the rate and orientation of both cell division and cell growth. In addition, the cell division cycle is tightly coordinated with many aspects of plant cell physiology (Gutierrez, 2016).

The mechanistic aspects of the plant cell cycle and oriented divisions, and their links to environmental conditions or hormonal signals, have been covered by excellent recent reviews (Livanos and Müller, 2019; Desvoyes and Gutierrez, 2020; Shimotohno et al., 2021). Here, we discuss how cell cycle progression and oriented divisions are coordinated with tissue growth and cell fate decisions during plant organogenesis. Due to the breadth of the subject and space limitations, we can only present a selection of topics and results, based largely on work in *Arabidopsis thaliana*; accordingly, gene and protein names are from Arabidopsis unless stated otherwise. We summarize the basic concepts and players in plant cell cycle and division, and then focus on their links to cues from within each cell, such as metabolic state, cell size, cell geometry, and cell identity, and to cues from their surroundings, such as tissue mechanics and signals from neighboring cells. We aim to provide a useful framework to understand the crucial interplay of cell autonomous and intercellular control of the frequency and orientation of cell divisions, and to highlight the main challenges that we face ahead in this field.

## Universal features of the cell cycle

The cell division cycle consists of a series of highly regulated processes that end up in the production of two daughter cells. The four well-known cell cycle phases are the S-phase, when the genome is duplicated; the mitotic (M) phase, when the genetic material and the cytoplasmic components are segregated to the daughter cells; and the gap phases G1 and G2 prior to S and M, respectively (Harashima et al., 2013; Gutierrez, 2016). This definition combines two temporally separated cycles: the “genome replication” and the “chromosome segregation” cycles, and was established long ago through observation of mitotic figures and pulses of radioactive nucleoside analogs in bean (*Vicia faba* L.) root cells (Howard and Pelc, 1953).

When other processes with a cyclic behavior in proliferating cells are considered, the occurrence of multiple cycles becomes apparent. Thus, the rise and fall of cyclin-dependent kinase (CDK) activity that drives much of the cell cycle gives rise to the “CDK cycle”, which depends on the accumulation of activator cyclins and precedes the “transcriptional cycles” of mid–late G1 and late G2. Other molecular networks with a cyclic pattern are detectable but less understood, e.g. the cycle of histone and chromatin modification. Additionally, most cellular components double their amount in rhythm with the cell cycle (D'Ario and Sablowski, 2019).

In addition to its multiple interlocked cycles, a universal feature of the eukaryotic cell cycle is that it is unidirectional (Coudreuse and Nurse, 2010). Several redundant pathways ensure that, except for exceptional cases, once a cell has progressed up to a certain point in the cell cycle, e.g. S-phase, it will not be able to return to a previous stage, e.g. G1. This unidirectional flow is enforced by complementary mechanisms, including transcriptional regulation, changes in subcellular localization, targeted proteolysis and post-translational modification of regulatory factors. Conserved, evolutionarily ancient mechanisms underlie the universal features of the eukaryotic cell cycle. However, some regulatory features differ among eukaryotic lineages, including plants (Harashima et al., 2013).

## The plant cell cycle

As in other eukaryotes (Coudreuse and Nurse, 2010), CDK activity is believed to be the core determinant of cell cycle progression in plants. The main plant CDKs are CDKA, which is equivalent to the canonical Cdc2 of fission yeast (Nowack et al., 2012) and functions throughout the cell cycle, and the plant-specific CDKBs, which are active in G2/M. CDK activity depends on cyclin subunits, which change at different stages of the cell cycle. It is reasonable to assume that both qualitative and quantitative differences in CDK nature and activity drive the cell cycle in plants (Nowack et al., 2012). A specific feature of plant cyclins is their diversity, with over 50 homologs in Arabidopsis (Shimotohno et al., 2021). This diversity is believed to reflect the variety of environmental inputs that affect plant cell cycle progression, and specialized cyclin functions in different cell types (see below).

**Figure 1:**
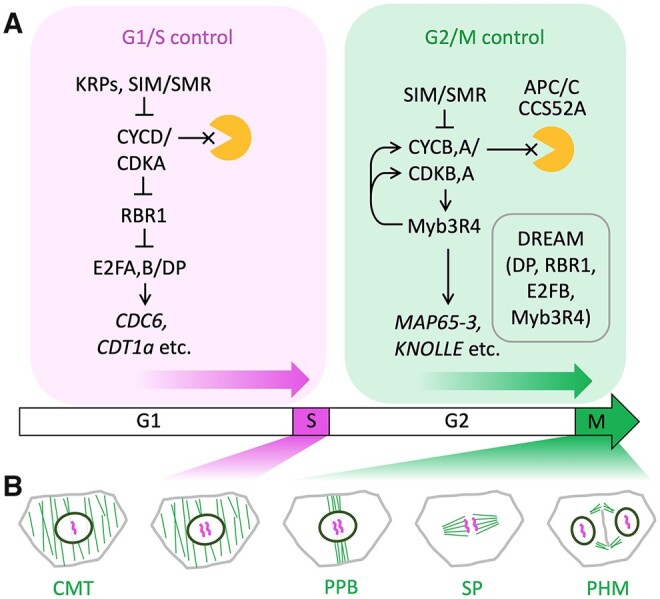
Simplified regulatory pathways and cytoskeletal changes in the plant cell cycle. A, The wide arrow (bottom) represents progression through the cell cycle phases (G1, S, G2, M); above, cell cycle regulators mentioned in the main text are placed in simplified pathways that control the G1/S (magenta) and G2/M (green) transitions; blunted lines and arrows represent inhibitory and activating interactions; lines ending in a cross indicate degradation by the proteasome (orange shape); the box within G2/M shows components of the putative plant DREAM complex. B, Cytoskeletal changes required for chromosome segregation and cell division: microtubules (green) are re-organized from the CMT arrays seen in G1, S, and G2, to the PPB, mitotic spindle (SP), and phragmoplast (PHM); chromosomes (magenta) are replicated in S-phase (magenta shading) and segregated in mitosis (green shading); the PPB anticipates the orientation of the SP, PHM and, consequently, of the new cell wall separating the daughter cells.

The G1 and G2 phases are not merely gap stages for S and M, respectively. Instead, a series of processes, frequently linked to chromatin dynamics (Desvoyes et al., 2014), are key to successful progression to the next phase (Figure 1). In this section we focus on the main processes occurring in each phase of the plant cell cycle, highlighting features that are relevant to subsequent sections and directing the reader to comprehensive reviews for mechanistic details.

### G1-phase

The first nuclear event detectable after mitosis is chromatin decondensation. The increased chromatin accessibility in early G1 has been connected with the acquisition of cell fate, as illustrated at the single locus level by the *GLABRA2* (*GL2*) gene, which determines trichoblast and atrichoblast root cell types. In atrichoblasts, but not in trichoblasts, the *GL2* locus becomes highly accessible in late mitosis and early G1 (Costa and Shaw, 2006), allowing high *GL2* expression and increased levels of histone H3 acetylation (Caro et al., 2007). At the genome-wide level, gene loops and topologically associating domains present in the mother cell may be maintained or modified with the acquisition of a new cell fate. It will be important to explore how this chromatin reorganization could occur (Sequeira-Mendes and Gutierrez, 2016), given that plants lack the CTCF (CCCTC-Binding Factor) proteins that help in the process in animal cells (Shoaib et al., 2020). It is worth noting the recent proposal that some maize transcription factors, such as members of the TCP, AP2-EREB, and LBD families, might act as functional equivalents of animal CTCF (Marand et al., 2021).

Assembly of pre-replication complexes (pre-RCs) is another crucial event in early G1, necessary to specify DNA replication origins (ORIs) that will fire in S-phase. Formation of pre-RC requires the low CDK levels of early G1 and occurs by successive assembly of a conserved multisubunit complex containing Origin Recognition Complex, Cell Division Control 6 (CDC6), Chromatin Licensing and DNA Replication Factor 1 (CDT1), and the Minichromosome Maintenance helicase; Shultz et al., 2007; Riera et al., 2017; Ocaña-Pallarès et al., 2020). Pre-RC assembly occurs during the same time window when chromatin becomes accessible for cell fate decisions, and stimulation of *GL2* expression by CDT1 suggests a link between both processes (Caro et al., 2007; Caro and Gutierrez, 2007).

G1 progression is restricted by the repressive function of the RETINOBLASTOMA-RELATED 1 (RBR1) protein (Figure 1). The transcriptional repression by RBR1 is maintained by low levels of D-type cyclins and high levels of CDK inhibitors, such as Kip-related proteins (KRPs; Desvoyes and Gutierrez, 2020), which together modulate G1 duration (Han et al., 2021). Multiple RBR1 phosphorylation events, mediated by CYCD/CDKA or by other kinases, such as S6K1 (see below), relieve E2F/DP transcription factors. These activate expression of a large set of target genes (Ramirez‐Parra et al., 2003; Vandepoele et al., 2005; Naouar et al., 2009), whose promoters contain RBR1 (Bouyer et al., 2018) and encode proteins required in S-phase or later in G2 (Figure 1).

### S-phase

Genome replication, including both DNA and chromatin components, is initiated in S-phase by the activation (firing) of thousands of ORIs scattered across the genome (Siddiqui et al., 2013). There is a strict timing whereby certain genomic regions consistently replicate in early, mid, or late S-phase (Wear et al., 2017; Concia et al., 2018). Genome-wide maps of ORIs showed that a large fraction of ORIs colocalize with chromatin regions of high accessibility (Wheeler et al., 2020), mostly in proximal promoters and the 5′-end of genes (Sequeira-Mendes et al., 2019). The timing of DNA replication and likely ORI specification depend on chromatin features, as demonstrated for H4K20 methylation in animal cells (Tardat et al., 2010). The plant-specific H3K27me1 mark, deposited by ARABIDOPSIS TRITHORAX-RELATED PROTEIN 5 (ATXR5) and ATXR6 in heterochromatin regions, is required to prevent re-replication (Jacob et al., 2010) and reduces the DNA replication-transcription conflicts in heterochromatin (Hale et al., 2016), underscoring the importance of coordinating these two processes during S-phase.

DNA methylation at cytosines (mC) can occur in various forms (mCG, mCGH, and mCHH) and varies during the cell cycle, in particular during S-phase, due to the separation of parental DNA strands as the replication fork progresses. While mCG and mCGH are maintained in replicating cells, mCHH is selectively lost, a dynamic with implications for changes in cell fate (Borges et al., 2021). Chromatin reconstitution past the DNA replication fork depends on the chaperone-mediated deposition of histones to form nucleosomes onto newly synthesized DNA and on the function of histone modifying enzymes (Loppin and Berger, 2020; Probst et al., 2020). Differences in the local chromatin landscape in daughter strands could be explored as another mechanism for fate decisions that would complement the events occurring in early G1.

### G2-phase

Chromosome segregation during mitosis needs to be an exact process, otherwise it may lead to chromosomal aberrations and loss of large genomic regions. As a consequence, a G2 checkpoint has evolved to assess the completeness of genome replication and its integrity. Activation of this checkpoint leads to a transient G2 arrest that allows time for repair of DNA and chromatin using highly coordinated pathways (Shimotohno et al., 2021). In case of irreparable damage, plant cells have evolved a strategy to skip mitosis by switching to the endocycle (see below), thus avoiding the risk of segregating damaged chromosomes.

Progression during G2 and to the G2/M transition requires CDK activity that is mainly dependent on B-type cyclins (Figure 1). In yeast and animal cells, a positive feedback loop through the Cdc25 phosphatase increases Cdk activity during the G2/M progression (Harashima et al., 2013; Umeda et al., 2019). Arabidopsis, however, lacks Cdc25 and instead relies on a transcriptional wave that reinforces CDK activity through expression of G2/M cyclins (Kobayashi et al., 2015; Ning et al., 2020) and activates genes required later in M-phase, such as those encoding the syntaxin KNOLLE (Lukowitz et al., 1996) or the *Arabidopsis thaliana* MICROTUBULE-ASSOCIATED PROTEIN 65-3 (MAP65-3; Müller et al., 2004) and ENDOSPERM DEFECTIVE1 (EDE1; Pignocchi et al., 2009). This transcriptional wave is regulated by a multiprotein complex comparable to the animal DREAM (DP, Rb-like, E2F, and MuvB) complex (Kobayashi et al., 2015). Plant DREAM complexes identified so far include those containing RBR1 and the transcription factors *Arabidopsis thaliana* PUTATIVE c-MYB-LIKE TRANSCRIPTION FACTOR MYB3R4 (MYB3R4) and E2F TRANSCRIPTION FACTOR B (E2FB), activators of G2/M gene expression, or MYBR3,5 and E2FC, which maintain the repression of G2/M genes in cells that have exited the cell cycle (Kobayashi et al., 2015; Magyar et al., 2016; Desvoyes and Gutierrez, 2020). Further work will be needed before the full set of plant DREAM complexes and their constituents is identified.

The time spent in G2 also depends on the cell status during organogenesis. Cells undergoing their last cell cycle undergo a longer G2-phase (Otero et al., 2016), likely needed for the extensive remodeling of their chromatin landscape and transcriptional networks required to initiate differentiation. It is worth noting that an extended G2-phase also occurs in the last cell cycle during animal organogenesis (Blythe and Wieschaus, 2015).

Another prominent feature of G2/M progression is the reconfiguration of the microtubule (MT) cytoskeleton, which plays central roles in chromosome segregation and cell division (Figure 1). During most of the plant cell cycle, MT arrays are organized near the cell’s surface, forming the cortical MT (CMT) array, which guides the deposition of cellulose microfibrils in the cell wall, and consequently influences the rate and orientation of cell wall extension during cell growth (Paredez et al., 2006). At the end of G2, the CMT is reorganized into the pre-prophase band (PPB), which forms a narrow band of MTs together with F-actin, usually wrapped around the mid-section of the cell (Livanos and Müller, 2019). The PPB is a transient structure, which is believed to leave behind a mark in the cell cortex to guide the orientation of the mitotic spindle and of the subsequent cell division during cytokinesis (Schaefer et al., 2017; Livanos and Müller, 2019). Until recently, apart from the transcriptional control of *MAP65-3* and *EDE1* by MYB3R proteins (Kobayashi et al., 2015), not much was known about the coordination between cell cycle progression and the sequential re-organization of MT arrays; a recent pre-print, however, provided evidence that cyclins of the *CYCB1* family function redundantly to regulate MT dynamics during the cell cycle (Motta et al., 2021).

### M-phase and cytokinesis

The M-phase consists of two processes that run in parallel: chromosome segregation and physical separation of the two daughter cells (cytokinesis). Chromosome condensation follows a massive phosphorylation of specific residues of histone proteins by AURORA kinases (Desvoyes et al., 2014; Shoaib et al., 2020). The centromeres of condensed chromosomes associate with MTs of the mitotic spindle during metaphase (Figure 1). This association is mediated by the kinetochores, multiprotein complexes that also play a crucial role in supervising the correct attachment of the spindle fibers to chromosomes (Motta and Schnittger, 2021); unattached kinetochores activate the spindle assembly checkpoint, which delays progression to anaphase (Komaki and Schnittger, 2017). The metaphase/anaphase transition is determined by an abrupt decrease of CDK activity due to rapid degradation of mitotic cyclins by the anaphase promoting complex/cyclosome (APC/C; Saleme et al., 2021).

Later in anaphase and telophase, the spindle MTs reorganize to form the phragmoplast (PHM; Figure 1), which directs formation of the cell plate, a plant-specific structure produced by fusion of vesicles originating from the trans-Golgi network and containing cell wall components. In most cases, the cell plate starts at the central position of the dividing cell and expands centrifugally toward the mother cell wall, finally separating the daughter cells (Buschmann and Müller, 2019), although the occurrence of cell plates formed centrifugally is not universal (Cutler and Ehrhardt, 2002). The composition and integrity of the cell wall are important for cell cycle progression, as revealed by the changes in the expression levels of several cyclins after reduction in the activity of cellulose biosynthesis (Gigli-Bisceglia et al., 2018).

The M processes described above can be bypassed, leading to repeated rounds of DNA replication without chromosome segregation (endocycles; Breuer et al., 2014; Edgar et al., 2014). In cells that differentiate as polyploid cells, such as trichomes and sepal giant cells in Arabidopsis, the shift from mitotic to endocycles is promoted during G2 by CDK inhibitors of the SIAMESE-RELATED (SMR) family (Churchman et al., 2006; Roeder et al., 2010). SMR proteins bind and inhibit both CDKA and CDKB (Kumar et al., 2015). Another way to promote the shift to endocycles is through premature degradation of G2/M cyclins (Cebolla et al., 1999), as seen in the Arabidopsis root tip, where cytokinin induces the expression of *CELL CYCLE SWITCH PROTEIN 52 A1* (*CCS52A1*) (Takahashi et al., 2013), which encodes a protein that targets cyclin CYCA2;3 for APC/C-mediated degradation (Imai et al., 2006; Boudolf et al., 2009).

## Coordination of cell cycle with metabolism

The cell cycle steps described above are energy-intensive and accompanied by extensive biosynthetic activity (e.g. DNA replication, translation, new cell wall synthesis). In addition, in tissues where cells proliferate while maintaining their average size, the time between divisions needs to match the time to double the amount of all cellular components. All of this requires coordination between cell cycle progression and metabolism.

Target of Rapamycin (TOR) is a phosphatidylinositol 3-kinase-related protein kinase that integrates metabolic signals to regulate growth and cell division across eukaryotes. In conditions favorable to growth, TOR is activated and promotes anabolism, whereas under energy- and nutrient deficiency, TOR inhibition promotes catabolism (Burkart and Brandizzi, 2021). As in animals and yeast, levels of glucose and amino acids are important in the regulation of TOR activity, but in plants it remains unclear how information about these metabolite levels is conveyed to TOR (Burkart and Brandizzi, 2021). In line with its role in linking metabolic status to the cell cycle, TOR is preferentially expressed in meristems (Menand et al., 2002; Barrada et al., 2019). Induction of meristem cell proliferation by glucose or sucrose requires TOR, and phosphorylation of E2FA and E2FB by TOR in vitro revealed a direct link to G1/S control (Xiong et al., 2013; Li et al., 2017). A phosphoproteomics screen in Arabidopsis cell culture did not confirm the phosphorylation of E2FA/B in vivo, but did show TOR-dependent phosphorylation of RBR1, which is likely mediated by the TOR target S6K1 (Henriques et al., 2010; Van Leene et al., 2019). Consistent with this, treatment with a TOR inhibitor leads to accumulation of cells in G1 (Desvoyes et al., 2020). Another confirmed in vivo target of TOR is the YEAST YAK1-RELATED GENE 1 kinase, which was shown to function downstream of TOR and regulate the expression of SMR CDK inhibitors (Barrada et al., 2019; Forzani et al., 2019). SMRs have been implicated in the control of both G1/S and G2/M transitions, so in addition to the well-supported link to G1–S control through the RBR1/E2F pathway, the TOR pathway may also regulate the progression to mitosis (Ahmad et al., 2019).

## Coordination between cell cycle and cell size

Under favorable conditions, metabolism will sustain the steady macromolecular synthesis that results in cell growth. The consequent increase in cell size has important effects on cellular function (D'Ario and Sablowski, 2019). For example, if the overall shape of the cell remains the same, surface area will not increase in proportion to cell volume, affecting the cells’ ability to exchange nutrients and signals. As detailed below, the surface to volume ratio also affects the mechanical properties of the cell and the tissues where it is embedded (Bassel et al., 2014; Sapala et al., 2018). Thus, similar to yeast and mammalian cells, at least some plant cell types have evolved mechanisms to stay within a given size range by linking cell cycle progression to cell size (D'Ario and Sablowski, 2019).

The link between cell size and cell cycle has been demonstrated in the shoot apical meristem. The asymmetric cell divisions and heterogeneous cell growth rates seen in the meristem should increase cell size variability, yet meristem cell sizes remain relatively constant over long periods of proliferation (Uyttewaal et al., 2012; Serrano-Mislata et al., 2015). Computer simulations, experiments using recovery from cell size perturbation and quantitative analyses of time-course images all supported the existence of a cell-autonomous feedback between cell size and cell cycle progression (Serrano-Mislata et al., 2015; Willis et al., 2016; Jones et al., 2017).

**Figure 2:**
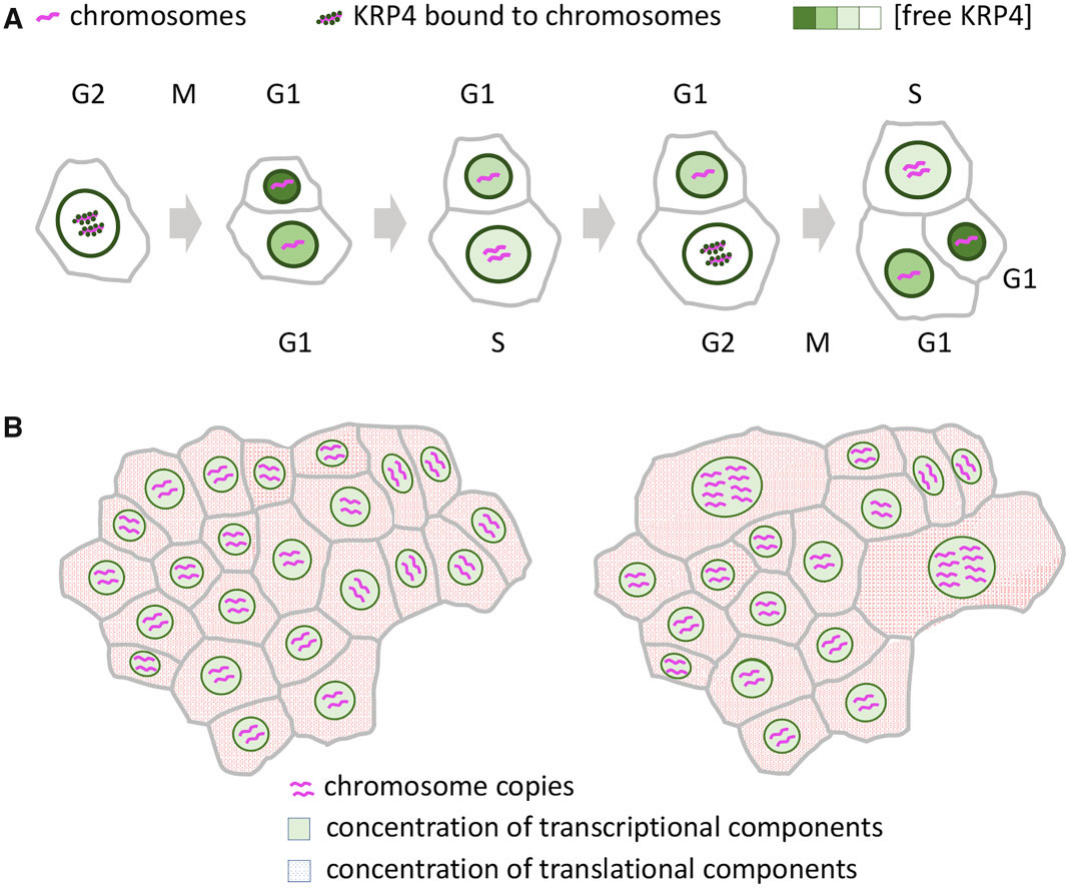
Coordination between cell cycle and cell size. A, Control of G1 length by size at birth in meristem cells. The KRP4 protein (green) associates with chromosomes (magenta) during mitosis and is released in equal amounts in the daughter cells; cells born small spend more time growing in G1 until KRP4 is diluted sufficiently for progression to S-phase, and consequently cell size differences are corrected by the time cells reach S-phase. B, Relation between cell size and ploidy in the sepal epidermis. Cells that undergo endoreduplication (marked with asterisks on the left) continue to grow while skipping cell divisions; although the cells are larger, their relative growth rate and the concentration of DNA and biosynthetic machinery are comparable to what they would be if the cells had continued to divide (asterisks on the right); in this way, growing tissues maintain a comparable amount of genome copies, regardless of whether they are packaged in diploid or polyploid cells.

A proposed molecular mechanism links G1–S progression to cell size, using chromatin content as a molecular scale: during mitosis, daughter cells inherit the same amount of the CYCD/CDKA inhibitor KRP4, bound to mitotic chromosomes; consequently, after asymmetric divisions the smaller cell has a higher initial concentration of KRP4 and needs to grow more before KRP4 is diluted sufficiently to allow the transition to S-phase (D'Ario et al., 2021; Figure 2A). Analogous mechanisms may operate in mammalian and yeast cells, albeit using different regulators of the G1/S transition (Swaffer et al., 2020; Zatulovskiy et al., 2020). It will be interesting to explore to what extent the use of DNA content as a size scale can explain the connection between cell size and ploidy (see below), and more generally with genome size.

Outside the meristems, cell size is much more variable, in part because specialized cells have their own characteristic size range, such as stomatal guard cells (GCs) compared to leaf epidermal pavement cells. Another reason for increased variability in cell volumes is cell expansion driven by increase vacuolar size, which probably evolved as a metabolically economical way to achieve rapid organ growth (D'Ario and Sablowski, 2019). If cells assess their size not based on their total volume, but on their cytoplasmic or nuclear contents, then the coupling to cell cycle progression would be obscured in cells with large vacuoles.

A further reason for size heterogeneity is that a subset of cells can undergo endoreduplication, leading to ploidy-related increases in cell size (Robinson et al., 2018; Lang and Schnittger, 2020). However, the increased size caused by endoreduplication is not universal and was not seen, for example, in Arabidopsis mesophyll cells (Katagiri et al., 2016). Endocycles occur as part of the differentiation of specific cell types, such as trichomes and giant cells in Arabidopsis sepals (Roeder et al., 2010; Lang and Schnittger, 2020), or can occur stochastically, without being a necessary feature of the cell type, as seen in epidermal pavement cells (Kawade and Tsukaya, 2017). Considering that endoreplication can be induced by DNA damage, likely as a mechanism to allow continued somatic growth in conditions that disrupt mitosis (Ramirez-Parra and Gutierrez, 2007b; Adachi et al., 2011), DNA damage responses might also contribute to stochastic shifts to endocycles.

The reason why polyploid cells tend to be larger remains unclear. It has often been argued that increased ploidy sustains the growth of cells with high metabolism, implying that DNA template is biochemically limiting (Lang and Schnittger, 2020). Support for this idea came, for example, from the higher transcription rate seen in polyploid cells of tomato (*Solanum lycopersicum*) fruits (Bourdon et al., 2012). However, if cell size increased in proportion to ploidy and the amount of biosynthetic machinery were proportional to cell size, then the concentration of DNA template relative to the transcriptional machinery should remain unchanged in polyploid cells (Figure 2B). Accordingly, sepal cells of different ploidy have similar relative growth rates, and consequently the number of genome copies per sepal area remains the same regardless of the balance between mitotic and endocycles (Roeder et al., 2010; Tauriello et al., 2015). Thus, changes in ploidy would not be expected to enhance overall gene expression per unit of tissue mass. However, ploidy may differentially affect a subset of genes, including cell wall-related genes, consistent with the frequent association of endocycles with rapid cell expansion (Bhosale et al., 2019) and with ploidy-related changes in cell wall composition (Corneillie et al., 2019). The connection between ploidy and expression of cell wall genes might reflect the reduced surface to volume ratio of larger cells, as seen in yeast (Wu et al., 2010), and changes in cell mechanics, as larger cells are subject to more mechanical stress (Bassel et al., 2014; Sapala et al., 2018).

## Coordination between cell division and cell shape

**Figure 3:**
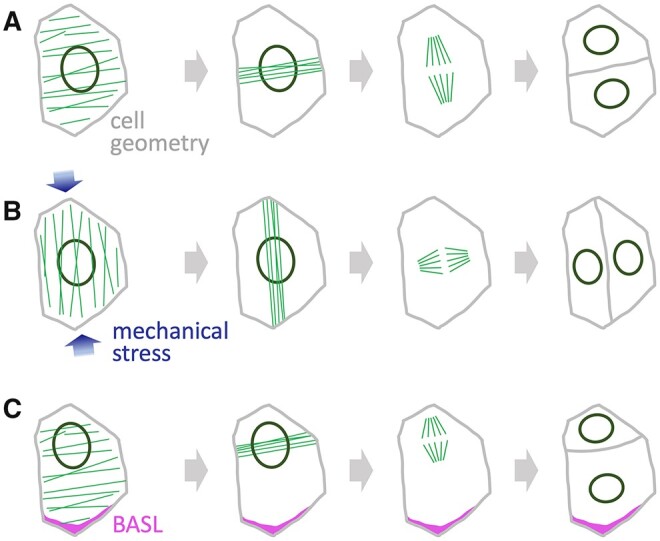
Selection of the cell division plane. A, In the absence of external cues, the division plane depends primarily on cell geometry; interactions between MT arrays (green) and the nucleus tend to select the smallest plane that divides the cell approximately in half. B, Mechanical stress, for example caused by growth of interconnected cells, leads to alignment of MTs with the stress orientation (blue arrows), influencing the position of the PPB and the subsequent division. C, Polarity proteins, such as BASL (magenta), activate mechanisms that re-position the nucleus, consequently changing the PPB position and the cell division plane.

Cell growth frequently involves change not only in cell size, but also shape. While size is linked to the timing of cell cycle progression, shape affects the orientation of cell divisions. When cells proliferate without attributing distinct fates to sister cells, divisions tend to be symmetrical. In these cases, the orientation of divisions is well explained by the classic rule that a new wall is placed in the shortest available path that divides the cell in half (Errera, 1886), with more recent refinements including a probabilistic element (Besson and Dumais, 2011). To correctly predict division planes based on 3D cell shapes from Arabidopsis embryos, it has also been necessary to assume that the new plane passes close to the centroid of the mother cell (Moukhtar et al., 2019). In physical terms, these rules are consistent with a mechanism that selects the shortest path from the nucleus to the cortical MT array (Lloyd, 1991; Asada, 2019; Livanos and Müller, 2019), which is reorganized in the vicinity of the nucleus into the PPB that stabilizes the selected division plane (Schaefer et al., 2017; Figure 3).

Computer models have also demonstrated how MT arrays could respond consistently to cell geometry and yet be very responsive to even a very slight bias used to represent external cues (Chakrabortty et al., 2018a; Mirabet et al., 2018), and the predicted effects of cell geometry on MT arrays have been elegantly confirmed in confined cells with enforced geometry (Durand-Smet et al., 2020). Thus, the dynamics of MT arrays are central to understanding how the cell division plane depends on both cell geometry and external signals.

One important external cue that influences MT arrays is mechanical stress, as shown by the effect of externally applied stress on the orientation of cortical MT arrays (Hamant et al., 2008; Robinson and Kuhlemeier, 2018) and of cell divisions (Lintilhac and Vesecky, 1984). These responses require a constant re-organization of arrays through MT polymerization and destruction. This dynamic behavior requires severing of MTs by katanin and de-stabilization of MTs by the NIMA-RELATED KINASE 6 (NEK6) kinase; in mutants for either, the stabilized MT arrays become less responsive to mechanical stimuli (Uyttewaal et al., 2012; Takatani et al., 2020). How MTs align to the orientation of mechanical stress on the cell wall, however, remains a major unanswered question (Hamant et al., 2019; Robinson, 2021).

External signals also control asymmetric cell divisions that are associated with the establishment of different cell fates, as seen in the formation of the epidermal layer during embryogenesis or in the divisions that create the spatial arrangement of different cell types during stomatal development (Geisler et al., 2000; Shpak et al., 2005; Cartwright et al., 2009; Yoshida et al., 2014). In the embryo, auxin signaling promotes asymmetric divisions, which deviate from the “minimum area” rule (Yoshida et al., 2014). The underlying mechanism remains unknown, but simulations of MT arrays in realistic embryo cell shapes provided useful clues: the effect of auxin could be reproduced by assuming localized stabilization of MTs at cell edges and along specific cell walls (Chakrabortty et al., 2018b).

During stomatal development, the EPIDERMAL PATTERNING FACTOR 1 (EPF1) peptide functions as an intercellular signal in a mechanism that ensures the optimal positioning of GCs by controlling the orientation of asymmetric divisions that produce different cell types during stomatal development (see below; Hara et al., 2007; Dong et al., 2009). Within the recipient cell, these asymmetric divisions depend on polarly localized proteins such as the BREAKING OF ASYMMETRY IN THE STOMATAL LINEAGE (BASL; Dong et al., 2009; Houbaert et al., 2018). BASL affects the cell division plane by promoting MT-mediated repositioning of the nucleus, which as mentioned above, interacts with the cortical MT array to position the PPB and the subsequent division plane (Muroyama et al., 2020; Figure 3).

## Coordination with tissue and organ growth

As cells are connected both physically and through chemical signals, decisions on when and how to divide are not made in isolation. We next describe examples of how these decisions are coordinated with the cell’s neighbors, either in the immediate vicinity or within the whole organ where the cells are embedded.

Live imaging has shown that both in the shoot meristem and in developing organs, neighboring cells show considerable variability in their rates of growth and division (Uyttewaal et al., 2012; Hong et al., 2016). At first sight, this would appear problematic for cells that need to collectively produce organs of a predictable size and shape. However, evidence has accumulated that this heterogeneous behavior is actually an important part of achieving robust morphogenesis (Hong et al., 2016; Hervieux et al., 2017; Fruleux and Boudaoud, 2019). For example, sepals of the Arabidopsis *ftsH protease 4* (*ftsh4*) mutant showed more uniform cellular growth rates, but more variable organ size and shape (Hong et al., 2016). These defects are caused by disruption of a mitochondrial protease and the consequent accumulation of reactive oxygen species (ROS), suggesting that ROS constrains the ability of cells to adjust their growth rates. This local flexibility is likely required during tissue growth to avoid the buildup of mechanical stresses that could disrupt morphogenesis. In line with this idea, irregular growth of tissues and organs is also caused by mutation of katanin or NEK6, which as mentioned above maintain the MT dynamics required for normal responses to mechanical stress (Uyttewaal et al., 2012; Takatani et al., 2020).

Reflecting the propagation of mechanical forces through interconnected cell walls, cellular responses to mechanical stress are coordinated across tissues, as seen in the MT alignment in hypocotyls in response to endogenous or externally applied forces (Hejnowicz et al., 2000; Robinson and Kuhlemeier, 2018), or in the alignment of MT arrays along organ boundaries (Hamant et al., 2008). The MT alignment not only guides the reinforcement of tissues through the pattern of cellulose microfibril deposition, but also influences the orientation of cell divisions, which match stress orientation at organ boundaries or under external compression (Lintilhac and Vesecky, 1984; Hamant et al., 2008; Louveaux et al., 2016). Additionally, mechanical forces coordinate the positioning of cell polarity proteins, as seen in the leaf epidermis for BREVIS RADIX-LIKE 2 (BRXL2; Bringmann and Bergmann, 2017). However, unlike mechanical effects on cell division and on the orientation of cellulose deposition, the tissue-wide orientation of BRXL2 and BASL is independent of MT arrays (Mansfield et al., 2018). Superimposed on the tissue-wide coordination, local chemical signaling through EPF1 may override both mechanical cues (Bringmann and Bergmann, 2017) and the tissue-wide cue, similar to the way auxin signaling appears to override geometrical–mechanical cues for cell division orientation.

The alignment of BASL/BRXL2 with the orientation of mechanical stress poses a riddle: mechanical stress has orientation but not direction (i.e. it can be represented by lines or double arrows, but not by simple arrows), but BASL/BRXL2 accumulate with a specific direction along the predicted lines of stress, suggesting that BASL localization also depends on a tissue-wide polarity field (Mansfield et al., 2018). SOSEKI (SOK) proteins have emerged as candidate components of this polarity field: SOK proteins localize to specific cell edges, and different SOK family members are oriented along different organ axes (Yoshida et al., 2019). Polar SOK localization is stable, depends on cell wall integrity but not on MTs, and requires oligomerization through a conserved domain that functions similarly in proteins involved in planar polarity in animals (van Dop et al., 2020). Intriguingly, overexpression of *SOK1* disrupted cell division orientation (Yoshida et al., 2019; van Dop et al., 2020), but the links between SOK proteins and the cellular machinery that orients cell division remain unknown. A further caveat is that SOK localization and function have been studied mostly in embryos and roots, so links to the work on BASL/BRXL2 depend on confirmation that SOKs also function in leaf development.

The local adjustment of oriented divisions is not the only compromise between processes within individual cells and across tissues. While meristem dimensions generally mirror the size and number of its component cells, determinate organs such as leaves can accommodate considerable variation in cell size and numbers within a relatively constant final size (Hemerly et al., 1995; De Veylder et al., 2001; Ramirez-Parra and Gutierrez, 2007a; Hisanaga et al., 2015). This implies that the size of determinate organs primarily depends on processes that affect cellular growth, which can be partitioned into smaller or larger cells by changes in cell cycle progression—as also shown in animals by classic experiments using sectors with loss of Cdk1 or E2F function in Drosophila imaginal disks (Weigmann et al., 1997; Neufeld et al., 1998).

The production of heterogeneous cell sizes by flexible deployment of the cell cycle is shown clearly in the sepal epidermis, where the spatial pattern of giant cells reflects a temporal pattern of shifts from mitotic cycles to endocycles, without changes in growth rates (Roeder et al., 2010). There is, however, a limit to how much cell cycle regulation can be adjusted without affecting the overall organ growth. This appears to be true in particular for G1/S progression, as illustrated by the *jagged* mutant, in which floral organ growth is inhibited to a large extent due to activation of *KRP* genes (Schiessl et al., 2014); in other words, the number of genome copies can become a limiting factor for tissue growth.

## Coordination with cell fate

Tissue and organ growth rely on the orchestrated organization of various cell types with a characteristic anatomy and physiology. Cells with distinct fates are often generated by asymmetric (formative) divisions and therefore cell cycle factors are targets of developmental signals to coordinate cell production with cell fate. We next discuss examples of tight links between cell cycle and cell fate.

### Quiescent center

**Figure 4:**
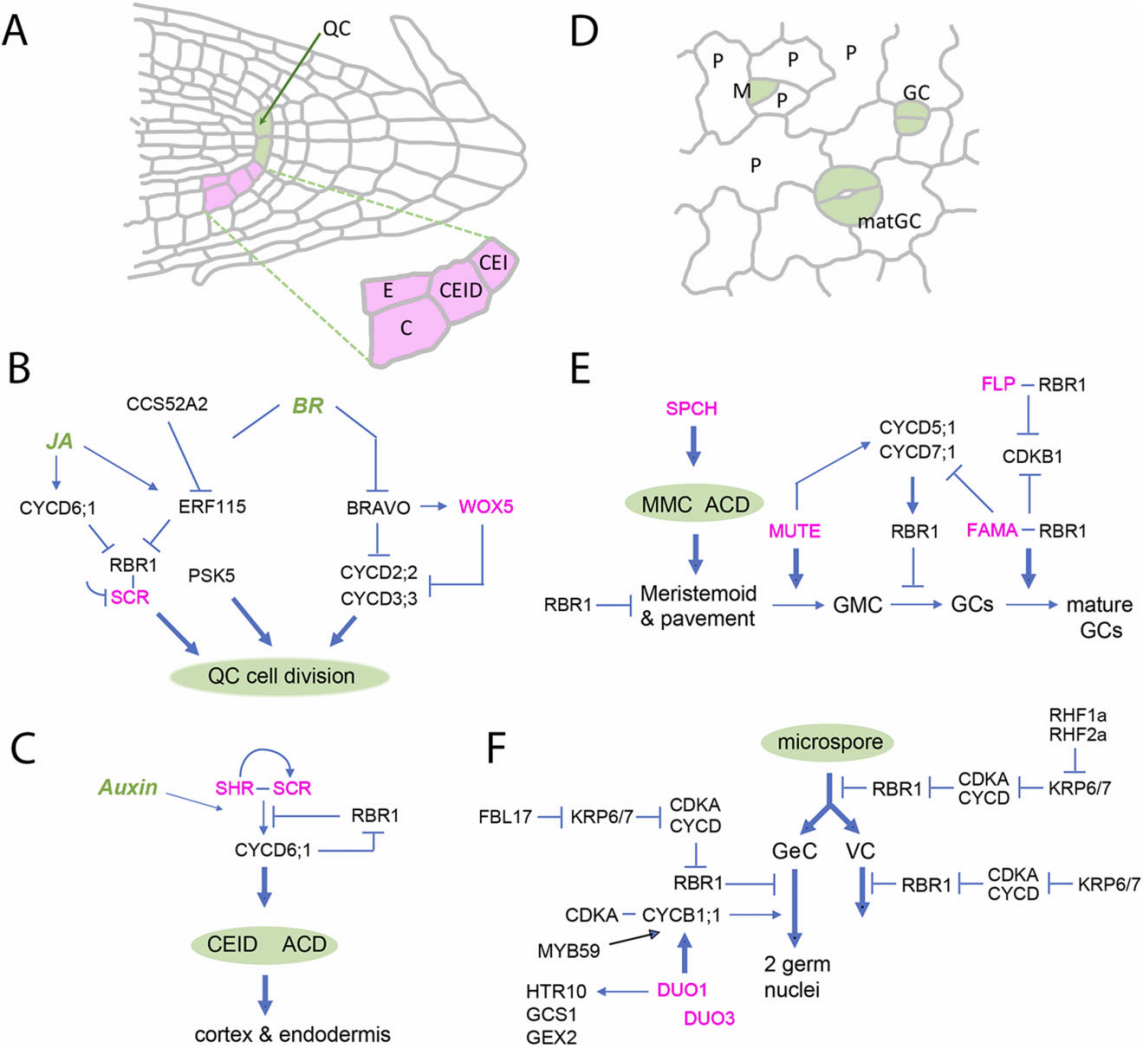
Coordination between cell division and cell fate. A, Cell outlines in the Arabidopsis root meristem, indicating the position of specific cell types: the QC is marked in green; magenta marks the CEI, the CEID and the endodermis (E) and cortex (C) cells that result from asymmetric division of the CEID. B, C, Schematic relationships of cell fate genes (magenta) and the cell cycle machinery in the QC (B) and cortex-endodermis (C); JA: jasmonic acid. D, Cell outlines in the developing cotyledon epidermis, with stomatal lineage cells marked in green: the meristemoid (M) divides asymmetrically to renew itself and produce the surrounding stomatal lineage ground cells; the meristemoid eventually changes identity and becomes a guard mother cell, then divides symmetrically to produce two GC, which differentiate into the mature guard cells of a functional stoma. E and F, Schematic relationships of cell fate genes (magenta) and the cell cycle machinery in the stomatal lineage (E) and male gametophyte (F). In B, C, E, and F: thick arrows highlight the final step in cell fate specification, blunted lines and arrows represent inhibitory and activating interactions, respectively, and hormonal signals are marked in green.

The quiescent center (QC), identified at the tip of the root apical meristem long ago (Clowes, 1953; Dubrovsky and Barlow, 2015), consists of a group of a few cells that rarely divide under normal conditions (Figure 4A). The QC cells are the source of signals that organize the root meristem (van den Berg et al., 1997), and are believed to function as long-term backup cells that are activated to replenish the root stem cell niche after wounding or DNA damage (Xu et al., 2006; Cruz-Ramírez et al., 2013; Heyman et al., 2013; Vilarrasa-Blasi et al., 2014).

The low division frequency of QC cells depends on the R2R3 MYB transcription factor BRAVO (Vilarrasa-Blasi et al., 2014; Figure 4B). BRAVO is highly expressed in QC and vascular stem cells, and directly represses the expression of genes necessary for cell cycle progression (e.g. *CYCD3;2*). In addition, BRAVO activates the QC cell fate WOX5, which in turn represses several cell cycle activators (Vilarrasa-Blasi et al., 2014). Conversely, brassinosteroids (BRs) stimulate QC cell division (González-García et al., 2011) through at least two complementary pathways (Figure 4B). On one side, BR represses *BRAVO* expression (Vilarrasa-Blasi et al., 2014). On another, BR transcriptionally stimulates the expression of *ERF115*, a member of the large family of ethylene response factors (Heyman et al., 2013). ERF115 triggers QC cell division by signaling through the PSK5 and PSKR1 phytosulfokine peptides, known regulators of root growth and cell proliferation (Kutschmar et al., 2009). ERF115 also mediates recovery of the root meristem after wounding by promoting QC cell division (Figure 4B), a process dependent on transcriptional activation of ERF115 by jasmonic acid signaling (Zhou et al., 2019).

QC identity not only affects cell cycle progression, but also depends on cell cycle regulators. SCARECROW (SCR), a GRAS family transcription factor that specifies QC and ground tissue cells (Di Laurenzio et al., 1996), directly interacts with RBR1 through an LxCxE motif, similar to the motif that mediates the interaction of RBR1 with D-type cyclins and E2F transcription factors (Cruz-Ramírez et al., 2012). ERF115 and RBR1 also physically interact, relieving the inhibitory effect of RBR1 on SCR, although this is not mediated by the LxCxE motif also found in ERF115 (Zhou et al., 2019). Together, these data show an intimate link between cell fate and cell cycle progression in the root meristem.

### Cortex and endodermis cell lineages

These two root cell types, known together as ground tissue, originate from two consecutive asymmetric cell divisions (ACDs): first, a stem cell, the cortex/endodermis initial (CEI), located in the distal part of the root tip in contact with QC cells, divides asymmetrically and anticlinally to renew itself and produce a CEI daughter (CEID) cell. Then, the CEID divides asymmetrically along the longitudinal axis of the root, forming two cells that eventually differentiate into cortex and endodermis cells (Figure 4A). Both the second ACD and acquisition of the two cell fates depend on the coordinated function of several components of the cell cycle machinery and two transcription factors that regulate cell identity.

CEID cell fate specification depends on the GRAS transcription factors SCR and SHORTROOT (SHR; Di Laurenzio et al., 1996; Helariutta et al., 2000). RBR1 forms a repressor complex with the SHR-SCR heterodimer that prevents ACD (Figure 4C); in response to high auxin levels in the CEID, SHR/SCR independently activates the expression of *CYCD6;1* by directly binding to its promoter (Sozzani et al., 2010; Cruz-Ramírez et al., 2012). The Med31 subunit of the Mediator complex appears to be needed at this stage (Zhang et al., 2018). CYCD6;1 activates the CDKA;1 and CDKB1;1 kinases that phosphorylate RBR1 and release its repressor activity. The use of a hypomorphic *cdka;1* mutant and loss of function *cdkb1;1* mutants strongly suggests a sequential RBR1 phosphorylation by these two kinases (Cruz-Ramírez et al., 2012; Weimer et al., 2012), in a manner analogous to Rb phosphorylation in mammalian cells (although by different kinases and cyclins; Munro et al., 2012). Additionally, CYCA3;4 appears to activate a CDK, likely CDKA;1, necessary for ACD (Willems et al., 2020). Finally, SCR, RBR1, and CYCD6;1 are targeted for proteasome proteolysis in such a way that this regulatory module is reset after ACD (Cruz-Ramírez et al., 2012). Then, SCR is no longer expressed in the cortex lineage whereas its expression is maintained by SHR in the endodermis, establishing different fates in these two cell types. The participation of RBR1 in ACD underscores its role as a key regulator of formative divisions, also occurring in other settings (Desvoyes and Gutierrez, 2020) (see below).

### Stomatal lineage

The concerted activity of the SPEECHLESS (SPCH), MUTE, and FAMA transcription factors in the stomatal lineage is an excellent example of coordination of cell specification factors with cell cycle regulators in controlling the occurrence of both symmetrical and asymmetrical divisions (Desvoyes and Gutierrez, 2020).

Two GCs make up the stoma in the leaf epidermis of Arabidopsis and are formed after a complex series of cell divisions coordinated with acquisition of specific cell fates. The process initiates with one ACD of the protodermal meristemoid mother cell (MMC) to give rise to a meristemoid and a stomatal lineage ground cell (Figure 4D). Another two ACDs result in a meristemoid surrounded by three pavement cells. Eventually, the meristemoid differentiates into a guard mother cell (GMC) that, by symmetrical division, produces two GCs (Han and Torii, 2016; Simmons and Bergmann, 2016). An excess of DNA replication initiator proteins, e.g. CDC6 or CDT1 (Castellano et al., 2001, 2004), or compromised RBR1 function (Park et al., 2005; Desvoyes et al., 2006; Borghi et al., 2010) leads to unrestricted proliferation of stomatal precursors and consequently an increase in stomatal number.

The ACD of meristemoid cells is triggered by SPCH (MacAlister et al., 2007; Lampard et al., 2008; Figure 4E). MUTE promotes symmetrical division of the GMC by upregulating *CYCD5;1*, and activates *CDKB1* together with *CYCD7;1*, thus inactivating the repressor RBR1 function (Han et al., 2018; Weimer et al., 2018). A recent pre-print reports that this symmetrical division is slower than the ACD as a result of MUTE-dependent activation of the CDK inhibitor SMR4, which extends G1 (Han et al., 2021). Members of the DREAM complex also control GMC symmetric division by a mechanism that tentatively involves RBR1 and the SOL1/SOL2 transcription factors (Simmons et al., 2019). Acquisition of GC fate and final differentiation requires the redundant function of RBR1-FAMA and RBR1-FLP (FOUR LIPS) complexes that inhibit *CYCD7;1* and *CDKB1* expression (Boudolf et al., 2004; Lee et al., 2014; Matos et al., 2014; Figure 4E). The coordination between cell cycle regulators and cell fate factors in the stomatal lineage has also been documented by single-cell transcriptomics (Lopez-Anido et al., 2021).

As described earlier in this review, in addition to specific interactions of cell cycle factors and cell fate regulators, ACD in the stomatal lineage depends on proteins with polarized subcellular localization such as BASL, which break the symmetry of cell division and establish functional differences between the daughter cells. This process is closely connected to cell fate specification: BASL and BRXL2 are direct targets of SPCH (Lau et al., 2014). BRI1 SUPPRESSOR 1-LIKE (BSL) protein phosphatases also interact with BASL to mediate the ACD of MMCs (Guo et al., 2021). BSL polarization occurs at the initiation of mitosis, although the mechanism of coordination with regulators of late stages of the cell cycle is not presently known.

### Gametophyte

Gametophyte development is another case of close links between cell fate acquisition and the cell cycle machinery. One example occurs during the transition between the diploid to haploid phases of the plant’s life cycle. In the developing ovule, a single MMC undergoes meiosis to generate the haploid megaspore, from which the female gametophyte develops. Selection of the MMC involves a switch from mitotic cycles to meiosis and a change in cell identity, including repression of the stem cell identity gene *WUSCHEL* (*WUS*), which is active in early ovule development. The RBR1 pathway connects both processes: KRP4, 6 and 7 inhibit progression of the mitotic cycle, while RBR1 also represses *WUS* in the MMC (Zhao et al., 2017).

KRPs and RBR1 also control cell division commitment in the male germline. The first division of the male microspore is asymmetric, resulting in one daughter cell with the cell cycle arrested (the vegetative cell, VC) while the other (the generative cell, GeC) divides again to generate the two sperm nuclei. In addition, VC and GeC acquire different epigenetic features (Berger and Twell, 2011; Borges et al., 2012; Peters et al., 2017; Ashapkin et al., 2019). The male microspore ACD is controlled by the RBR1 pathway and is triggered by proteasome-dependent degradation of, at least, KRP6 and KRP7, mediated by the RING-finger E3 ligases RHF1a and RHF2a (Liu et al., 2008; Zhao et al., 2012). The difference between VC and GeC relies on the KRP6/7 levels, which are maintained high in the VC, preventing its progression through the cell cycle (Figure 4F). Conversely, in the GeC, KRP6/7 are efficiently targeted for proteolysis by a SCF E3 ligase complex containing the F BOX-LIKE17 (FBL17) protein (Kim et al., 2008; Gusti et al., 2009). This favors the accumulation of CYCD, allowing the inactivation of the repressor function of RBR1 and promoting the G1/S transition. Later, the MYB R2R3 transcription factor DUO POLLEN 1 (DUO1, which is specifically expressed in the GeC, upregulates *CYCB1;1* (Rotman et al., 2005; Borg et al., 2011), which is required together with *CYCB1;2* for GeC division (Motta et al., 2021). Both DUO1 and DUO3 activate expression of other targets, e.g. the pollen-specific *HISTONE THREE RELATED 10* (*HTR10*; Brownfield et al., 2009). The intricacies of these regulatory pathways are fully supported by the phenotypes of *duo1*, *duo3*, *rbr1*, *fbl17*, and *cdka;1* mutants (Berger and Twell, 2011).

## Conclusions and perspectives

The examples described above highlight the main players that link the cell cycle with growth and cell fate in plants. The RBR1 pathway emerges repeatedly as a central nexus, with links to the TOR pathway, playing a role in the regulation of cell size and interactions with cell fate regulators in many of the best-studied plant developmental processes. This central role is a universal feature of the retinoblastoma pathway, which in animal cells also has a role in size control (Zatulovskiy et al., 2020) and in cell fate, for example, by directly repressing the stem cell factors Octamer-binding transcription factor 4 (Oct4) and Sex Determining Region Y-box 2 (Sox2) in mouse (Kareta et al., 2015). One feature that so far appears unique to plants, however, is the direct physical interaction between RBR1 and developmental regulators such as SCARECROW and FAMA. An important question for the future is how changes in cell fate might be connected to chromatin changes during the cell cycle, such as the increased chromatin accessibility in early G1 or the replication of chromatin marks during S-phase.

In the coordination of cell division orientation with cell growth and fate, MT dynamics have a prominent role, both through the links between the cortical MT array and the formation of the PPB, and in the mechanism that re-positions the nucleus before asymmetric divisions. Important unanswered questions in this area include how MTs align with the orientation of mechanical stress, how internal and external cues lead to positioning of the nucleus by MT- and actin-dependent mechanisms, and what molecular links connect auxin signaling, localized MT dynamics and division plane orientation. Another area that requires further investigation is how the re-organization of the MT arrays during the cell cycle is connected to the orderly changes in the activity of cell cycle regulators.

Identifying core, conserved mechanisms is not the only important aim. A future challenge is the identification of the cell cycle genes active in the context of a developing plant. This expectation is based on the fact that many of them are redundantly expressed in cultured cells but increasing evidence demonstrates their cell type- or cell fate-specific pattern. The question is whether different cells use common or unique cell regulatory toolkits. Furthermore, the processes discussed here have been studied mostly in Arabidopsis; little is known about their diversity during plant evolution. Revealing both conservation and diversity of the cellular basis of plant morphogenesis will be essential to understand how the rich variety of plant shapes is generated, and from a practical point of view, to increase our ability to modify crop growth in useful ways.

### Accession numbers

For reference, all proteins mentioned in this review are listed in Supplemental Data Set S1 with their full names, abbreviations, and accession numbers.

## Supplemental data 

The following materials are available in the online version of this article.

**Supplemental Data Set S1**. List of all genes and proteins mentioned in this review.

## Supplementary Material

koab222_Supplementary_Data

## Notes

R.S. and C.G. contributed equally at all stages of writing the manuscript; R.S. produced Figures 1–3, and 4, A and D; C.G. produced Figure 4, B, C, E, and F; both authors contributed to improving all figures.

The authors responsible for distribution of materials integral to the findings presented in this article in accordance with the policy described in the Instructions for Authors (https://academic.oup.com/plcell) are: Robert Sablowski (robert.sablowski@jic.ac.uk) and Crisanto Gutierrez (cgutierrez@cbm.csic.es).
