# Supplementary material for: Cycling in a crowd: Coordination of plant cell division, growth, and cell fate
Source: Plant Cell. 2021 Sep 8;34(1):193–208. doi: 10.1093/plcell/koab222 (PMC8774096; doi:10.1093/plcell/koab222)
Supplement: koab222_Supplementary_Data [file koab222_supplementary_data.pdf]

SUPPLEMENTAL DATA

Supplemental Data Set S1: List of all genes and proteins mentioned in this review

Protein	Gene name	Full gene name	Organism	Accession number
ATRX5	*ATRX5*	*ARABIDOPSIS TRITHORAX-RELATED PROTEIN 5*	*Arabidopsis thaliana*	AT5G09790
ATRX6	*ATRX6*	*ARABIDOPSIS TRITHORAX-RELATED PROTEIN 5*	*Arabidopsis thaliana*	AT5G24330
BASL	*BASL*	*BREAKING OF ASYMMETRY IN THE STOMATAL LINEAGE*	*Arabidopsis thaliana*	AT5G60880
BRAVO	*BRAVO*	*BRAVO*	*Arabidopsis thaliana*	AT5G17800
BRXL2	*BRXL2*	*BREVIS RADIX-LIKE 2*	*Arabidopsis thaliana*	AT3G14000
CCS52A1	*CCS52A1*	*CELL CYCLE SWITCH PROTEIN 52 A1*	*Arabidopsis thaliana*	AT4G22910
CDC6	*CDC6*	*CELL DIVISION CONTROL 6*	*Arabidopsis thaliana*	AT2G29680
CDKA;1	*CDKA;1*	*CYCLIN DEPENDENT KINASE A 1*	*Arabidopsis thaliana*	AT3G48750
CDKB1;1	*CDKB1;1*	*CYCLIN DEPENDENT KINASE B 1;1*	*Arabidopsis thaliana*	AT3G54180
CDT1	*CDT1*	*Chromatin Licensing and DNA Replication Factor 1*	*Arabidopsis thaliana*	AT2G31270
CYCA2;3	*CYCA2;3*	*CYCLIN A2;3*	*Arabidopsis thaliana*	AT1G15570
CYCA3;4	*CYCA3;4*	*Cyclin A3;4*	*Arabidopsis thaliana*	AT1G47230
CYCB1;1	*CYCB1;1*	*CYCLIN B1;1*	*Arabidopsis thaliana*	AT4G37490
CYCB1;2	*CYCB1;2*	*CYCLIN B1;2*	*Arabidopsis thaliana*	AT5G06150
CYCD3;3	*CYCD3;3*	*CYCLIN D3;3*	*Arabidopsis thaliana*	AT3G50070
CYCD5;1	*CYCD5;1*	*CYCLIN D 5;1*	*Arabidopsis thaliana*	AT4G37630
CYCD6;1	*CYCD6;1*	*CYCLIN D6;1*	*Arabidopsis thaliana*	AT4G03270
CYCD7;1	*CYCD7;1*	*CYCLIN D 7;1*	*Arabidopsis thaliana*	AT5G02110
DUO1	*DUO1*	*DUO POLLEN 1*	*Arabidopsis thaliana*	AT3G60460
		DUO POLLEN 3	*Arabidopsis thaliana*	AT1G64570
E2FB	*E2FB*	*E2F TRANSCRIPTION FACTOR B*	*Arabidopsis thaliana*	AT5G22220
E2FC	*E2FC*	*E2F TRANSCRIPTION FACTOR C*	*Arabidopsis thaliana*	AT1G47870
EDE1	*EDE1*	*ENDOSPERM DEFECTIVE 1*	*Arabidopsis thaliana*	AT2G44190
EPF1	*EPF1*	*EPIDERMAL PATTERNING FACTOR 1*	*Arabidopsis thaliana*	AT2G20875
ERF115	*ERF115*	*ETHYLENE RESPONSE FACTOR 115*	*Arabidopsis thaliana*	AT5G07310
FAMA	*FAMA*	*FAMA*	*Arabidopsis thaliana*	AT3G24140
FBL17	*FBL17*	*F BOX-LIKE17*	*Arabidopsis thaliana*	AT3G54650
FLP	*FLP*	*FOUR LIPS*	*Arabidopsis thaliana*	AT1G14350
FTSH4	*FTSH4*	*FTSH PROTEASE 4*	*Arabidopsis thaliana*	AT2G26140
GL2	*GL2*	*GLABRA2*	*Arabidopsis thaliana*	AT1G79840
HTR10	*HTR10*	*HISTONE THREE RELATED 10*	*Arabidopsis thaliana*	AT1G19890
JAG	*JAG*	*JAGGED*	*Arabidopsis thaliana*	AT1G68480
KN	*KN*	*KNOLLE*	*Arabidopsis thaliana*	AT1G08560

KRP4	*KRP4*	*KIP-RELATED PROTEIN 4*	*Arabidopsis thaliana*	AT2G32710
KRP6	*KRP6*	*KIP-RELATED PROTEIN 6*	*Arabidopsis thaliana*	AT3G19150
KRP7	*KRP7*	*KIP-RELATED PROTEIN 7*	*Arabidopsis thaliana*	AT1G49620
MAP65-3	*MAP65-3*	*ARABIDOPSIS THALIANA MICROTUBULE-ASSOCIATED PROTEIN 65-3*	*Arabidopsis thaliana*	AT5G51600
MUTE	*MUTE*	*MUTE*	*Arabidopsis thaliana*	AT3G06120
MYB3R3	*MYB3R3*	*ARABIDOPSIS THALIANA PUTATIVE C-MYB-LIKE TRANSCRIPTION FACTOR MYB3R-3*	*Arabidopsis thaliana*	AT3G09370
MYB3R4	*MYB3R4*	*ARABIDOPSIS THALIANA PUTATIVE C-MYB-LIKE TRANSCRIPTION FACTOR MYB3R-4*	*Arabidopsis thaliana*	AT5G11510
MYB3R5	*MYB3R5*	*ARABIDOPSIS THALIANA PUTATIVE C-MYB-LIKE TRANSCRIPTION FACTOR MYB3R-5*	*Arabidopsis thaliana*	AT5G02320
NEK6	*NEK6*	*NIMA (NEVER IN MITOSIS, GENE A)-RELATED KINASE 6*	*Arabidopsis thaliana*	AT3G44200
Oct_4	*Oct_4*	*Octamer-binding transcription factor 4*	*Mus musculus*	MGI:101893
PSK5	*PSK5*	*PHYTOSULFOKINE 5 PRECURSOR*	*Arabidopsis thaliana*	AT5G65870
PSKR1	*PSKR1*	*PHYTOSULFOKIN RECEPTOR 1*	*Arabidopsis thaliana*	AT2G02220
RBR1	*RBR1*	*RETINOBLASTOMA-RELATED 1*	*Arabidopsis thaliana*	AT3G12280
RHF1a	*RHF1a*	*RING-H2 GROUP F1A*	*Arabidopsis thaliana*	AT4G14220
RHF2a	*RHF2a*	*RING-H2 GROUP F2A*	*Arabidopsis thaliana*	AT5G22000
SCR	*SCR*	*SCARECROW*	*Arabidopsis thaliana*	AT3G54220
SHR	*SHR*	*SHORTROOT*	*Arabidopsis thaliana*	AT4G37650
SMR4	*SMR4*	*SIAMESE-RELATED4*	*Arabidopsis thaliana*	AT5G02220
SOL1	*SOL1*	*SUPPRESSOR OF LLP1 1*	*Arabidopsis thaliana*	AT1G71696
SOL2	*SOL2*	*SUPPRESSOR OF LLP1 2*	*Arabidopsis thaliana*	AT4G14770
Sox2	*Sox2*	*Sex Determining Region Y-box 2*	*Mus musculus*	MGI:98364
SPCH	*SPCH*	*SPEECHLESS*	*Arabidopsis thaliana*	AT5G53210
WUS	*WUS*	*WUSCHEL*	*Arabidopsis thaliana*	AT2G17950
YAK1	*YAK1*	*YEAST YAK1-RELATED GENE 1*	*Arabidopsis thaliana*	AT5G35980
